# Considering parental hearing status as a social determinant of deaf population health: Insights from experiences of the "dinner table syndrome"

**DOI:** 10.1371/journal.pone.0202169

**Published:** 2018-09-05

**Authors:** Wyatte C. Hall, Scott R. Smith, Erika J. Sutter, Lori A. DeWindt, Timothy D. V. Dye

**Affiliations:** 1 Obstetrics & Gynecology and Clinical & Translational Science Institute, University of Rochester Medical Center, Rochester, New York, United States of America; 2 Office of the Associate Dean of Research, National Technical Institute for the Deaf, Rochester Institute of Technology, Rochester, New York, United States of America; 3 National Center for Deaf Health Research, University of Rochester Medical Center, Rochester, New York, United States of America; 4 Deaf Wellness Center, University of Rochester Medical Center, Rochester, New York, United States of America; 5 Pediatrics and Public Health Sciences, University of Rochester Medical Center, Rochester, New York, United States of America; TNO, NETHERLANDS

## Abstract

The influence of early language and communication experiences on lifelong health outcomes is receiving increased public health attention. Most deaf children have non-signing hearing parents, and are at risk for not experiencing fully accessible language environments, a possible factor underlying known deaf population health disparities. Childhood indirect family communication–such as spontaneous conversations and listening in the routine family environment (e.g. family meals, recreation, car rides)–is an important source of health-related contextual learning opportunities. The goal of this study was to assess the influence of parental hearing status on deaf people’s recalled access to childhood indirect family communication. We analyzed data from the Rochester Deaf Health Survey–2013 (n = 211 deaf adults) for associations between sociodemographic factors including parental hearing status, and recalled access to childhood indirect family communication. Parental hearing status predicted deaf adults’ recalled access to childhood indirect family communication (χ^2^ = 31.939, p < .001). The likelihood of deaf adults reporting “sometimes to never” for recalled comprehension of childhood family indirect communication increased by 17.6 times for those with hearing parents. No other sociodemographic or deaf-specific factors in this study predicted deaf adults’ access to childhood indirect family communication. This study finds that deaf people who have hearing parents were more likely to report limited access to contextual learning opportunities during childhood. Parental hearing status and early childhood language experiences, therefore, require further investigation as possible social determinants of health to develop interventions that improve lifelong health and social outcomes of the underserved deaf population.

## Introduction

Growing public health attention addresses underlying influences of early language acquisition and contextual learning experiences on lifelong health, such as the impact of the “30 million word gap” noted in children of low-income families [1]. One population for which early language experiences are particularly relevant is deaf children– the majority (more than 90%) of whom are born into hearing families [2] and cannot effortlessly access spoken language. Deaf children exposed to a natural sign language from birth are more likely to experience healthy, expected development than non-signing deaf children [3–6]. In contrast to deaf parents, hearing parents overwhelmingly do not sign with their deaf child [7–9]. The deaf population experiences significant health disparities–such as increased obesity, poorer mental health status (e.g., suicidal ideations, intimate partner violence, and interpersonal trauma), and increased use of the emergency departments, among others [10–13]. One possible underlying factor of these disparities and general deaf population health outcomes may be parental hearing status, moderated by parents’ developmental language and communication choices for their deaf child.

Chronic lack of accessible communication with hearing parents is a common childhood trauma reported by deaf adults [14]. Less than 8% of deaf children receive regular use of a natural sign language in fluent and bidirectional conversations [8]. Instead, common practice for deaf children’s language development is often cochlear implants without natural sign language exposure. Cochlear implant research with non-signing children continues to demonstrate highly variable speech and language outcomes–with most not achieving comparable results to their hearing peers in large scale studies [15–21]. In contrast, implanted children who sign from birth can demonstrate desired speech and language outcomes [22, 23].

These circumstances highlight that the majority of deaf children are at risk for not developing a native first-language foundation in either English or a natural sign language (i.e., American Sign Language). Delayed and/or absent exposure to an accessible first-language foundation is increasingly described as “language deprivation,” which may create risk for various developmental consequences (such as a “language deprivation syndrome” in some extreme cases) across the lifespan [7, 24–27]. One such developmental consequence is adult brain structure differences based on timing and quality of childhood language access [28–30].

This context of language deprivation risks helps to inform what Deaf epistemology describes as the *dinner table syndrome* [31]–a catch-all phrase to explain a commonly experienced phenomenon in the Deaf community of observing indirect auditory conversations and being unable to understand what is said, such as family discussions at the dinner table. Recent studies indicate that indirect family communication during childhood, such as family medical histories and general family health discussions, is an important source of health knowledge and literacy–domains in which many deaf adolescents and adults have gaps [32, 33].

Indirect family conversations include health-related contextual learning opportunities (e.g., “My mother has diabetes, her mother had a similar problem.”) that children often internalize as part of their adult health knowledge. As a result of not accessing health-related contextual learning opportunities (also described as incidental learning opportunities) [33], many deaf adolescents struggle with health vocabulary and knowledge–such as misconceiving cholesterol as something that might be added “to food to make it taste better” or something that actually “makes the heart pump better” [32]. Unfortunately, this appears to be a common phenomenon as many deaf adults are unable to describe the typical signs and symptoms of heart attack and stroke [34]. As a result, ongoing research is now focusing on specific dimensions of deaf people’s health knowledge and literacy (e.g., interactive health literacy) in order to identify some root causes for the relative poorer health of deaf people [33].

Access to indirect family communication is important for contextual learning opportunities, so it is not surprising that perceived ability to access parental communication is associated with deaf adolescents’ quality-of-life outcomes [4]. The dinner table syndrome phenomenon, situated in context of the broader risks of language deprivation, likely has a significant impact on health outcomes of deaf people. Therefore, just as socioeconomic status serves as a useful indicator for a set of environmental influences on childhood development, parental hearing status might be a comparable marker for deaf children’s lifelong health outcomes as partially represented here by access to contextual learning opportunities.

The aim of this study is to assess whether or not parental hearing status influences deaf adults’ recalled access to indirect family communication during childhood. We hypothesize that deaf adults with at least one deaf parent will report greater access to indirect family communication during childhood than deaf adults from families with hearing parents.

## Methods

The analyses used existing data from the Rochester Deaf Health Survey–2013 (RDHS-2013) developed and conducted by the Rochester Prevention Research Center: National Center for Deaf Health Research [35]. The University of Rochester IRB determined the RDHS-2013 to be surveillance and not research. RDHS-2013 includes items on parents’ hearing status and recalled access to indirect family communication during childhood.

Combining subject responses about their mother and father’s hearing status generated an “at least one deaf parent” variable. Recalled comprehension of indirect family communication responses were generated from a recoding of four responses (“most of the time,” “sometimes,” “a little,” never”) into a meaningful logical construct of two responses (“most of the time,” “sometimes to never”) for the following question:

“The next question will ask you about your experience communicating with your family when you were growing up as a child. So please think back to when you were younger than 18 years old. Family means your mother, father, sister or brother. It can mean your real parents, step parents, adopted parents or anyone you live with most. How often did you understand what your family members said when they were talking to each other (not directly to you) such as at the dinner table or in the living room?”

Analyses were performed in SPSS v23.0. Cochran’s test of conditional independence and Mantel-Haenszel’s test of common odds ratio estimate were performed to investigate possible sociodemographic and deaf-specific confounders. The resulting variables were entered into a logistic regression model to predict influence of parental hearing status on comprehension of indirect family communication. Possible confounders included in the analysis were common social and deaf-specific demographics (see Table 1) that were at least marginally (< .10) significant with both the predictor and outcome variables.

**Table 1:** Relationships between parental hearing status and other demographic factors with comprehension of indirect family communication.

		Comprehension of indirect family communication			
	Total sample	Most of the time	Sometimes to never		
	n = 211	n = 51 (24.2%)	n = 148 (70.1%)		
	n (col %)^a^	n (row %)	n (row %)	OR (95% CI)	p-Value
**Parental hearing status**					
Hearing	175 (82.9%)	34 (19.4%)	141 (80.6%)	17.6 (5.6–55.8)	0.01
At least one deaf parent	22 (10.4%)	17 (81.0%)	4 (19.0%)	Referent	
**Age**					
47 years or less	107 (50.7%)	31 (31.0%)	69 (69.0%)	0.6 (0.3–1.1)	0.08
48 years or more	104 (49.3%)	20 (20.2%)	79 (79.8%)	Referent	
**Gender**					
Male	90 (42.7%)	21 (41.7%)	62 (41.9%)	1.0 (0.5–2.0)	0.93
Female	121 (57.3%)	116 (58.3%)	86 (58.1%)	Referent	
**Race**					
Non-white	35 (16.6%)	11 (31.4%)	24 (68.6%)	1.4 (0.6–3.2)	0.39
White	161 (76.3%)	39 (24.4%)	121 (75.6%)	Referent	
**Mother education**					
H.S. or less	104 (49.3%)	27 (26.2%)	76 (73.8%)	1.0 (0.5–1.9)	0.98
Some college or more	91 (43.1%)	24 (26.4%)	67 (73.6%)	Referent	
**Father education**					
H.S. or less	94 (44.5%)	23 (24.7%)	70 (75.3%)	1.2 (0.6–2.2)	0.64
Some college or more	101 (47.9%)	28 (27.7%)	73 (72.3%)	Referent	
**Age of hearing loss onset**					
4 years or more	26 (12.3%)	8 (30.8%)	18 (69.2%)	1.3 (0.5–1.7)	0.58
3 years or less	169 (80.1%)	43 (25.6%)	125 (74.4%)	Referent	
**Have hearing aid**					
No	87 (41.2%)	21 (24.4%)	65 (75.6%)	0.9 (0.5–1.7)	0.65
Yes	110 (52.1%)	30 (27.3%)	80 (72.7%)	Referent	
**Have cochlear implant**					
No	162 (76.8%)	41 (25.5%)	120 (74.5%)	0.8 (0.4–1.8)	0.57
Yes	33 (15.6%)	10 (30.3%)	23 (69.7%)	Referent	

^a^ Respondents were not required to answer all survey questions so percentages may not total to 100%

## Results

Parental hearing status predicted recalled comprehension of indirect family communication (χ^2^ = 31.939, p < .001 with df = 1). Prediction success overall was 80.6% (33.3% for “most of the time” and 97.2% for “sometimes to never”). As seen in Table 1, having hearing parents increases the likelihood of reporting “sometimes to never” for recalled comprehension of indirect family communication by 17.6 times (Odds Ratio = 17.6, 95% CI: 5.6–55.8). No other sociodemographic or deaf-specific factor–including having hearing aids or cochlear implants–predicted these deaf adults’ recalled access to childhood indirect family communication, and were not included in the final regression model.

## Discussion

Childhood indirect family communication is a central component of contextual learning opportunities that influences adult health outcomes. Consequently, it is important to understand the indicators and circumstance that influence and fosters contextual learning so we can identify those at risk for poor lifelong health outcomes. Respondents’ recalled understanding of childhood indirect family communication was moderated by parental hearing status. This helps highlight the potential role parents of deaf children have in reducing current health disparities seen in the deaf population.

In particular, our findings exemplify the “dinner table syndrome” phenomenon that is a widespread experience for deaf people, but has yet to be studied analytically. From a public health standpoint, it is important to recognize that the sample reflects known proportions of parental hearing status and more than 90% of deaf children are born into hearing, typically non-signing households [9]; additionally, 80.1% of participants reported pre-lingual hearing loss onset at three years or younger. Consequently, it is possible the majority of deaf children are at risk for suboptimal access to contextual information beyond the current sample. Most importantly, parental hearing status and associated language developmental choices deserves more investigation as a useful predictive marker for both life outcomes of deaf people and as a critical target for future preventive interventions.

In this sample, having hearing aids and cochlear implants did not improve access to contextual learning opportunities for deaf adults who grew up in hearing families–although interpretation of this is limited because age of implantation and length of implant and hearing aid use was not asked. Regardless of whether adults in this sample may have gotten implants later in life, as only those under 30 would have been able to receive an implant in early childhood, this finding is similar to Smith and Samar (33) demonstrating the limited benefit of cochlear implants on deaf adolescents’ health literacy. All in all, these findings align with growing recognition of the risks of language deprivation and current limitations of using auditory intervention technology as a standalone approach (i.e., the common approach of cochlear implants with no natural sign language exposure), and requires more investigation.

While deaf parents are three times more likely to use sign language regularly at home with their deaf child than hearing parents [9], the positive impact on development likely goes further than just language exposure. For instance, a deaf child may feel less “different” or “left-out,” influencing their adult perceptions of inclusion and recalled access to indirect family communication. The known benefits of early sign language exposure do not have to be limited to a small percent of deaf children. Public health programs that incorporate deaf adult mentors can have positive outcomes for language and family well-being [36]. Further research on the role of having at least one deaf parent in a family and/or access to deaf adults would be useful for developing targeted strategies (such as a deaf mentor program) that holistically support hearing parents and family members in creating a healthy environment for the deaf child.

Overall attention to childhood language experiences should be a public health priority to improve the lifelong health outcomes of the deaf population. The findings of this study also highlight the important potential role of contextual learning for lifelong health outcomes in the public health literature. Other groups such as children from low SES families likely experience similar barriers, as represented by the “30 million word gap” phenomenon. Future public health research and interventions should include considerations of access to health-related contextual learning opportunities, especially for at-risk populations.

Our study is limited in that the RDHS-2013 recruitment methods mainly focused on outreach to deaf sign language users. No measure of childhood communication modalities (e.g., spoken language, sign language) was included and childhood experience was based on recall. Additionally, the variable of interest is one item from a cross-sectional survey. Future research should include additional items to assess a wider scope of contextual learning opportunities and elucidate more aspects of this complex phenomenon.

## Conclusion

Deaf individuals’ recalled access to indirect contextual learning opportunities is an important childhood experience moderated by their parents’ hearing status. Parental hearing status and associated developmental language choices needs further investigation as a social determinant of deaf population health. Public health research should focus on early childhood language and communication experiences of deaf children at risk for language deprivation and other at-risk populations to improve their lifelong health and social outcomes.

## References

[1] Greenwood CR, Carta JJ, Walker D, Watson-Thompson J, Gilkerson J, Larson AL, et al Conceptualizing a Public Health Prevention Intervention for Bridging the 30 Million Word Gap. Clin Child Fam Psychol Rev. 2017;20(1):3–24. 10.1007/s10567-017-0223-8 . 28150059

[2] Mitchell RE, Karchmer MA. Chasing the mythical ten percent: Parental hearing status of deaf and hard of hearing students in the United States. Sign Language Studies. 2004;4(2):138–63.

[3] Schick B, de Villiers P, de Villiers J, Hoffmeister R. Language and theory of mind: a study of deaf children. Child Dev. 2007;78(2):376–96. 10.1111/j.1467-8624.2007.01004.x . 17381779

[4] Kushalnagar P, Topolski TD, Schick B, Edwards TC, Skalicky AM, Patrick DL. Mode of communication, perceived level of understanding, and perceived quality of life in youth who are deaf or hard of hearing. J Deaf Stud Deaf Educ. 2011;16(4):512–23. 10.1093/deafed/enr015 ; PubMed Central PMCID: PMCPMC3202327. 21536686 PMC3202327

[5] Braden JP. An explanation of the superior performance IQs of deaf children of deaf parents. Am Ann Deaf. 1987;132(4):263–6. . 3442285 10.1353/aad.2012.0723

[6] Marshall C, Jones A, Denmark T, Mason K, Atkinson J, Botting N, et al Deaf children's non-verbal working memory is impacted by their language experience. Front Psychol. 2015;6:527 10.3389/fpsyg.2015.00527 ; PubMed Central PMCID: PMCPMC4419661. 25999875 PMC4419661

[7] Humphries T, Kushalnagar P, Mathur G, Napoli DJ, Padden C, Rathmann C, et al Language acquisition for deaf children: Reducing the harms of zero tolerance to the use of alternative approaches. Harm Reduct J. 2012;9:16 10.1186/1477-7517-9-16 ; PubMed Central PMCID: PMCPMC3384464. 22472091 PMC3384464

[8] Gallaudet Research Institute. Regional and national summary report of data from the 2009–10 annual survey of deaf and hard of hearing children and youth Washington, D.C.: Gallaudet University, 2011.

[9] Mitchell RE, Karchmer MA. Parental hearing status and signing among deaf and hard of hearing students. Sign Lang Stud. 2005;5(2):231–44.

[10] Barnett S, Klein JD, Pollard RQ Jr., Samar V, Schlehofer D, Starr M, et al Community participatory research with deaf sign language users to identify health inequities. Am J Public Health. 2011;101(12):2235–8. 10.2105/AJPH.2011.300247 ; PubMed Central PMCID: PMCPMC3222424. 22021296 PMC3222424

[11] Anderson ML, Leigh IW. Intimate partner violence against deaf female college students. Violence Against Women. 2011;17(7):822–34. 10.1177/1077801211412544 . 21676984

[12] Fellinger J, Holzinger D, Pollard R. Mental health of deaf people. The Lancet. 2012;379(9820):1037–44. 10.1016/S0140-6736(11)61143-4 . 22423884

[13] McKee MM, Winters PC, Sen A, Zazove P, Fiscella K. Emergency department utilization among deaf American Sign Language users. Disab Heal J. 2015;8(4):573–8. 10.1016/j.dhjo.2015.05.004 ; PubMed Central PMCID: PMCPMC4570852. 26166160 PMC4570852

[14] Anderson ML, Wolf Craig KS, Hall WC, Ziedonis DM. A Pilot Study of Deaf Trauma Survivors' Experiences: Early Traumas Unique to Being Deaf in a Hearing World. J Child Adolesc Trauma. 2016;9(4):353–8. 10.1007/s40653-016-0111-2 ; PubMed Central PMCID: PMCPMC5271372. 28138351 PMC5271372

[15] Kral A, Kronenberger WG, Pisoni DB, O'Donoghue GM. Neurocognitive factors in sensory restoration of early deafness: A connectome model. The Lancet Neurology. 2016;15(6):610–21. 10.1016/S1474-4422(16)00034-X . 26976647 PMC6260790

[16] Fink NE, Wang NY, Visaya J, Niparko JK, Quittner A, Eisenberg LS, et al Childhood Development after Cochlear Implantation (CDaCI) study: design and baseline characteristics. Cochlear Implants Int. 2007;8(2):92–116. 10.1179/cim.2007.8.2.92 . 17549807

[17] Niparko JK, Tobey EA, Thal DJ, Eisenberg LS, Wang NY, Quittner AL, et al Spoken language development in children following cochlear implantation. JAMA. 2010;303(15):1498–506. 10.1001/jama.2010.451 ; PubMed Central PMCID: PMCPMC3073449. 20407059 PMC3073449

[18] Tobey EA, Thal D, Niparko JK, Eisenberg LS, Quittner AL, Wang NY, et al Influence of implantation age on school-age language performance in pediatric cochlear implant users. Int J Audiol. 2013;52(4):219–29. 10.3109/14992027.2012.759666 ; PubMed Central PMCID: PMCPMC3742378. 23448124 PMC3742378

[19] Geers A, Brenner C, Davidson L. Factors associated with development of speech perception skills in children implanted by age five. Ear Hear. 2003;24(1 Suppl):24S–35S. 10.1097/01.AUD.0000051687.99218.0F . 12612478

[20] Davidson LS, Geers AE, Blamey PJ, Tobey EA, Brenner CA. Factors contributing to speech perception scores in long-term pediatric cochlear implant users. Ear Hear. 2011;32(1 Suppl):19S–26S. 10.1097/AUD.0b013e3181ffdb8b ; PubMed Central PMCID: PMCPMC3187573. 21832887 PMC3187573

[21] Tobey EA, Geers AE, Sundarrajan M, Lane J. Factors Influencing Elementary and High-School Aged Cochlear Implant Users. Ear Hear. 2011;32(1):27S–38S. 10.1097/AUD.0b013e3181fa41bb ; PubMed Central PMCID: PMCPMC3074604. 21499506 PMC3074604

[22] Hassanzadeh S. Outcomes of cochlear implantation in deaf children of deaf parents: comparative study. J Laryngol Otol. 2012;126(10):989–94. 10.1017/S0022215112001909 . 22906641

[23] Davidson K, Lillo-Martin D, Chen Pichler D. Spoken english language development among native signing children with cochlear implants. J Deaf Stud Deaf Educ. 2014;19(2):238–50. 10.1093/deafed/ent045 ; PubMed Central PMCID: PMCPMC3952677. 24150489 PMC3952677

[24] Hall WC. What You Don't Know Can Hurt You: The Risk of Language Deprivation by Impairing Sign Language Development in Deaf Children. Matern Child Health J. 2017;21(5):961–5. 10.1007/s10995-017-2287-y ; PubMed Central PMCID: PMCPMC5392137. 28185206 PMC5392137

[25] Hall ML, Eigsti IM, Bortfeld H, Lillo-Martin D. Auditory Deprivation Does Not Impair Executive Function, But Language Deprivation Might: Evidence From a Parent-Report Measure in Deaf Native Signing Children. J Deaf Stud Deaf Educ. 2017;22(1):9–21. 10.1093/deafed/enw054 ; PubMed Central PMCID: PMCPMC5189172. 27624307 PMC5189172

[26] Hall WC, Levin LL, Anderson ML. Language deprivation syndrome: a possible neurodevelopmental disorder with sociocultural origins. Soc Psychiatry Psychiatr Epidemiol. 2017:1–16. 10.1007/s00127-017-1351-7 . 28204923 PMC5469702

[27] Henner J, Caldwell-Harris CL, Novogrodsky R, Hoffmeister R. American Sign Language Syntax and Analogical Reasoning Skills Are Influenced by Early Acquisition and Age of Entry to Signing Schools for the Deaf. Front Psychol. 2016;7:1982 10.3389/fpsyg.2016.01982 ; PubMed Central PMCID: PMCPMC5183573. 28082932 PMC5183573

[28] Mayberry RI, Chen JK, Witcher P, Klein D. Age of acquisition effects on the functional organization of language in the adult brain. Brain and Language. 2011;119(1):16–29. 10.1016/j.bandl.2011.05.007 . 21705060

[29] Penicaud S, Klein D, Zatorre RJ, Chen JK, Witcher P, Hyde K, et al Structural brain changes linked to delayed first language acquisition in congenitally deaf individuals. Neuroimage. 2013;66:42–9. 10.1016/j.neuroimage.2012.09.076 . 23063844

[30] Skotara N, Salden U, Kugow M, Hanel-Faulhaber B, Roder B. The influence of language deprivation in early childhood on L2 processing: An ERP comparison of deaf native signers and deaf signers with a delayed language acquisition. BMC Neurosci. 2012;13:44 10.1186/1471-2202-13-44 ; PubMed Central PMCID: PMCPMC3404011. 22554360 PMC3404011

[31] Hauser PC, O'Hearn A, McKee M, Steider A, Thew D. Deaf epistemology: Deafhood and Deafness. Am Ann Deaf. 2010;154(5):486–92; discussion 93–6. . 20415284 10.1353/aad.0.0120

[32] Smith SR, Kushalnagar P, Hauser PC. Deaf adolescents' learning of cardiovascular health information: Sources and access challenges. J Deaf Stud Deaf Educ. 2015;20(4):408–18. 10.1093/deafed/env021 ; PubMed Central PMCID: PMCPMC4615750. 26048900 PMC4615750

[33] Smith SR, Samar VJ. Dimensions of Deaf/Hard-of-Hearing and Hearing Adolescents' Health Literacy and Health Knowledge. J Health Commun. 2016;21(sup2):141–54. 10.1080/10810730.2016.1179368 ; PubMed Central PMCID: PMCPMC5073377. 27548284 PMC5073377

[34] Margellos-Anast H, Estarziau M, Kaufman G. Cardiovascular disease knowledge among culturally Deaf patients in Chicago. Prev Med. 2006;42(3):235–9. 10.1016/j.ypmed.2005.12.012 . 16460789

[35] Barnett S, Matthews KA, Sutter EJ, DeWindt LA, Pransky JA, O'Hearn AM, et al Collaboration with deaf communities to conduct accessible health surveillance. Am J Prev Med. 2017;52(3S3):S250–S4. 10.1016/j.amepre.2016.10.011 . 28215374

[36] Watkins S, Pittman P, Walden B. The deaf mentor experimental project for young children who are deaf and their families. Am Ann Deaf. 1998;143(1):29–34. . 9557330 10.1353/aad.2012.0098

